# Chemical vapor deposition merges MoS_2_ grains into high-quality and centimeter-scale films on Si/SiO_2_†

**DOI:** 10.1039/d1ra06933k

**Published:** 2022-02-18

**Authors:** Mukesh Singh, Rapti Ghosh, Yu-Siang Chen, Zhi-Long Yen, Mario Hofmann, Yang-Fang Chen, Ya-Ping Hsieh

**Affiliations:** Department of Physics, National Taiwan University Taipei 106 Taiwan; Department of Physics, National Central University Chung Li 320 Taiwan; Institute of Atomic and Molecular Sciences, Academia Sinica Taipei 115 Taiwan yphsieh@gate.sinica.edu.tw; Molecular Science and Technology, Taiwan International Graduate Program, Academia Sinica Taipei 115 Taiwan

## Abstract

Two-dimensional molybdenum disulfide (MoS_2_) has attracted increasing attention due to its promise for next-generation electronics. To realize MoS_2_-based electronics, however, a synthesis method is required that produces a uniform single-layer material and that is compatible with existing semiconductor fabrication techniques. Here, we demonstrate that uniform films of single-layer MoS_2_ can be directly produced on Si/SiO_2_ at wafer-scale without the use of catalysts or promoters. Control of the precursor transport through oxygen dosing yielded complete coverage and increased connectivity between crystalline MoS_2_ domains. Spectroscopic characterization and carrier transport measurements furthermore revealed a reduced density of defects compared to conventional chemical vapor deposition growth that increased the quantum yield over ten-fold. To demonstrate the impact of enhanced scale and optoelectronic performance, centimeter-scale arrays of MoS_2_ photosensors were produced that demonstrate unprecedentedly high and uniform responsivity. Our approach improves the prospect of MoS_2_ for future applications.

## Abstract 2

Control of the precursor transport through oxygen dosing yields increased MoS_2_ coverage and increased connectivity between crystalline MoS_2_ domains.

## Graphical abstract

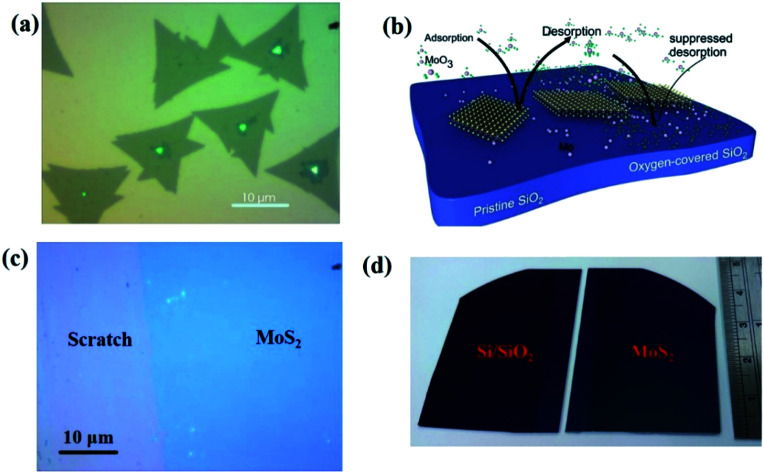

## Introduction

Molybdenum disulfide (MoS_2_) is a 2D material that has attracted the attention of researchers due to its unique electrical and optical properties.^1^ Based on its high carrier mobility,^2,3^ electrostatic controllability,^4^ environment stability,^5^ and thickness-induced bandgap tunability,^6,7^ MoS_2_ is considered an enabling material for future electronics.^8,9^ This promise is corroborated by the reported impressive performance of MoS_2_ in optoelectronic devices such as photodetectors,^10–15^ light emitters,^16,17^ and haptic sensors.^18^

However, to advance MoS_2_-based electronics from research into commercially viable applications, MoS_2_ production has to be compatible with existing, mature semiconductor fabrication technology. This requirement disqualifies mechanical exfoliation due to small crystallite size and unreliable placement. Moreover, synthesis on single-crystalline substrates such as sapphire,^19^ h-BN, mica^20–23^ and metal^24,25^ cannot be employed, despite their impressive performance, as the required large-scale and high-precision transfer step are beyond current capabilities. The most promising approach is the direct growth of MoS_2_ on silicon dioxide (SiO_2_) due to the experience of semiconductor industry with the material system^8,26^ and the existing commercial infrastructure. Unfortunately the growth on SiO_2_, to date, has only yielded discontinuous flakes compared to the uniform and continuous films grown on other substrates.^27–29^ Several approaches have been explored to overcome this challenge but they introduce other issues. First, the use of organic seeding materials like perylene-3,4,9,10-tetracarboxylic acid tetrapotassium salt (PTAS) can enhance the continuity of MoS_2_ but introduces impurities into the reactor and into the produced MoS_2_.^30,31^ Second, large grain sizes and continuous films can be achieved by liquid-substrate chemical vapour deposition (CVD) but salt-residue remains on the SiO_2_.^32^

In this study, we have introduced an approach to produce continuous single layer MoS_2_ at large-scale on Si/SiO_2_. This advance was accomplished by a modified CVD process that utilizes a small amount of oxygen. Using this method, high quality material with unprecedented continuity and uniformity could be synthesized, as evidenced by spectroscopic characterization and electron microscopy. The resulting material was applied to MoS_2_-based optoelectronic devices that demonstrate record-breaking performance and uniform properties throughout wafer-scale device array.

## Results and discussion

**Fig. 1:**
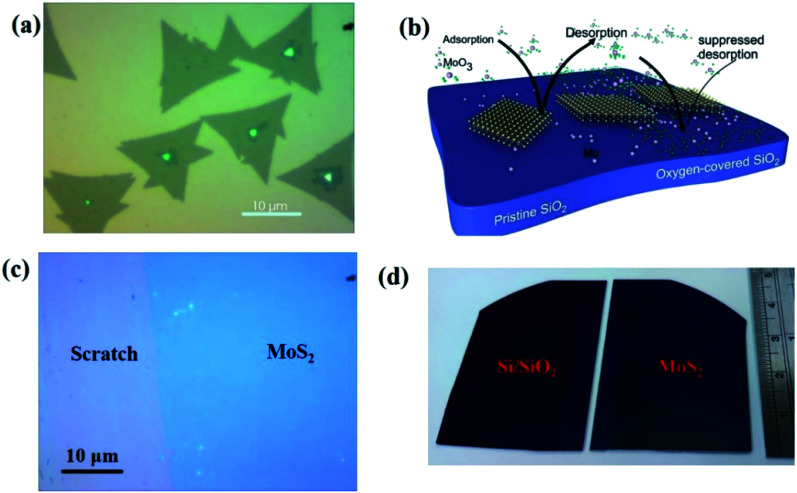
(a) Optical microscopy image of MoS_2_ films as deposited on Si/SiO_2_ substrate without O_2_. (b) Schematic of adsorption and desorption of gaseous phase MoO_3_ on Si/SiO_2_. (c) Optical microscopy image (taken after scratch the film gently by tweezer) of *in situ* O_2_ processed samples. (d) Camera image of wafer scale continuous MoS_2_ on Si/SiO_2_ (in right) and a blank Si/SiO_2_ substrate (in left) for the reference.

Chemical vapor deposition is an established process to synthesize MoS_2_ from gaseous precursors.^33–35^ We employ silicon oxide wafers as substrates and observe discontinuous triangular flakes with average dimensions of ∼10 μm size (Fig. 1(a)) when using established CVD parameters.^33,36^ Even long growth durations cannot connect these individual flakes, raising the question as to the limiting mechanism. The MoS_2_ growth by CVD is a multistep conversion process from gaseous MoO_3_ precursor into adsorbed species that are subsequently integrated into the outgrowing MoS_2_ crystal.^37^ The self-limiting grain size indicates the kinetic hindrance of precursor supply at long growth durations: at low MoS_2_ coverage, adsorption of MoO_3_ precursor proceeds quickly and flake growth occurs. At high MoS_2_ coverage, however, the adsorption efficiency decreases as less uncovered surface sites are available. Consequently, the MoS_2_ growth rate decreases until precursor adsorption and desorption are in equilibrium, and no further growth occurs (Fig. 1(b)).

**Fig. 2:**
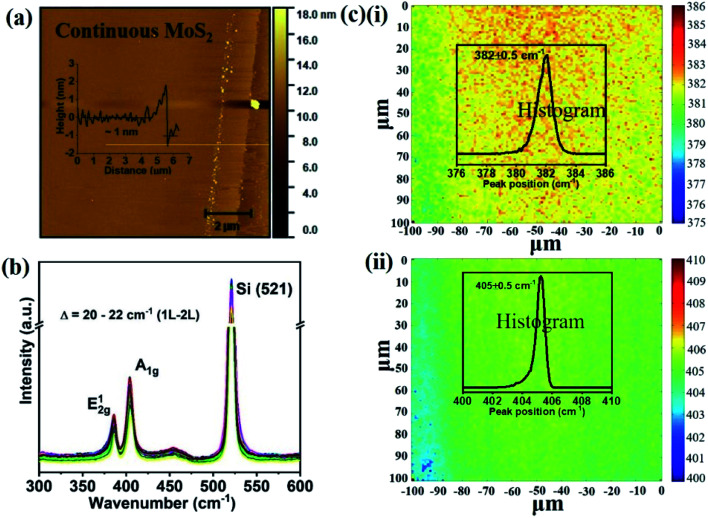
Characterization of *in situ* O_2_ processed continuous MoS_2_ films on Si/SiO_2_ (a) AFM image (scale bar: 2 μm) (height profile given in inset). (b) Raman spectra of the as deposited films measured at several randomly selected locations. (c) Raman mapping of (i) E^1^_2g_ and (ii) A_1g_ peak positions in 100 μm × 100 μm dimensions with 1 μm step size (histogram shown in inset).

**Fig. 3:**
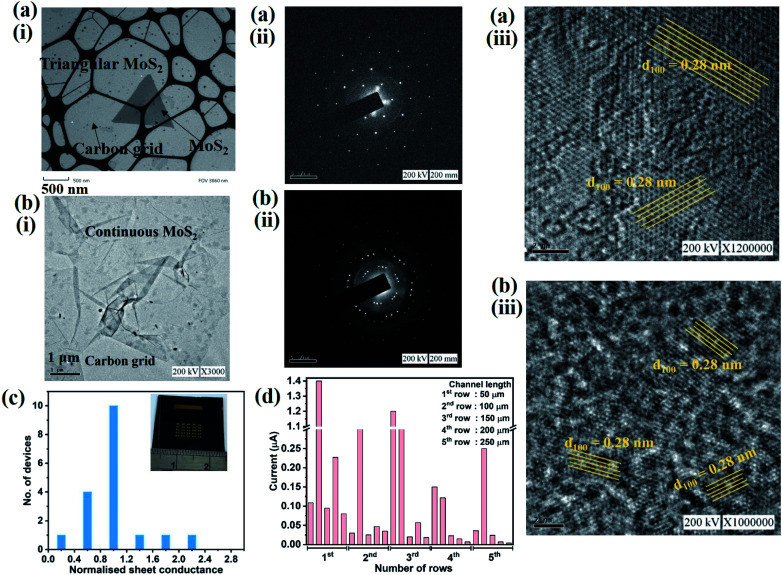
(a and b) TEM characterization of conventional (a) and oxygen-assisted (b) MoS_2_:(i) low resolution TEM image after transfer onto carbon-coated grid, (ii) SAED pattern (scale bar: 5 nm^−1^) (iii) HRTEM image (scale bar: 2 nm). Electrical characterization of uniformity: histogram of sheet conductance for 18 devices (c) in device array (see inset, (c)). Demonstration of channel continuity through current measurements within large device array of different channel lengths measured at fixed bias of 20 V in two terminal co-planar geometry.

We overcome this limitation by introducing 0.5 sccm of oxygen. Previous work has demonstrated that increased chemisorption of oxygen on silicon oxide surfaces stabilizes the MoO_3_ precursor^38^ and thus reduces the impact of precursor desorption on growth rate (Fig. 1(b)). Indeed, optical microscopy images demonstrate that the addition of oxygen during the CVD growth process produces continuous films (Fig. 1(c)). The modified oxygen-assisted CVD method can also grow uniform and continuous wafer-scale MoS_2_ (Fig. 1(d)). Detailed characterization of the oxygen-grown MoS_2_ by atomic force microscopy (AFM) corroborates the high uniformity of the film (Fig. 2a). The thickness of ∼1 nm presents a clear evidence of uniform single-layer MoS_2_ films^39,40^ (more details about the uniformity of the films can be found in the ESI S1†). This evaluation is confirmed by Raman spectra which show characteristic peaks at 382 ± 0.5 cm^−1^ and 404 ± 0.5 cm^−1^ corresponding to in-plane E^1^_2g_ mode and out-of-plane A_1g_ mode, respectively (Fig. 2b). These two peaks are well separated by 22 ± 0.5 cm^−1^, which indicates the single/bi layer nature MoS_2_ film.^39,41,42^ Raman mapping was employed to characterize the uniformity of the MoS_2_. Fig. 2c(i and ii) shows the spatial distribution of the E^1^_2g_ and A_1g_ peaks postion over a 100 × 100 μm^2^ area with a step size of 1 μm, demonstrating the absence of vacancies and the uniform peak separation of ∼22 ± 0.5 cm^−1^ throughout the film. TEM images (Fig. 3a(i) and b(i)) further corroborate the uniformity for both type of the films. The selected area electron diffraction (SAED) (Fig. 3a(ii) and b(ii)) and high-resolution transmission electron microscopy (HRTEM) (Fig. 3a(iii) and b(iii)) indicate the polycrystalline nature of O_2_-assisted MoS_2_ in comparison to conventional MoS_2_, where single crystal characteristic was observed. Apart from the optical characterizations, films were further characterized by electrical transport measurement in co-planar geometry for the centimetre scale area of MoS_2_ continuity. Fig. 3c and d represent the electrical sheet conductance and electrical current respectively, in two terminal co-planar geometry, for the device with different channel length of 50, 100, 150, 200 and 250 μm and at a fixed channel width of 580 μm. Each channel length belonging to one particular row and there are five row in the device. So, the data shown here is for 25 devices. The uniformity of current in MoS_2_ shows that films are continuous in centimetre scale. The current variation in each row indicates the polycrystalline nature of MoS_2_ films.

**Fig. 4:**
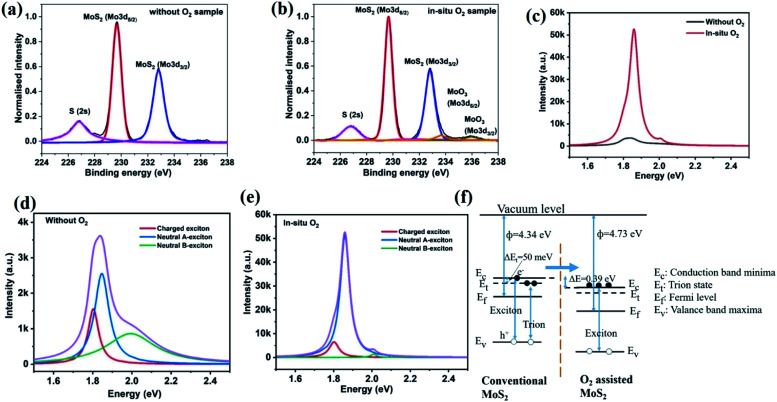
XPS spectra of Mo 3d binding energy for the films deposited (a) without O_2_ and (b) with *in situ* O_2_ samples. (c) PL spectra of single layer MoS_2_ for both the films; without O_2_ and with *in situ* O_2_. (d and e) Deconvoluted PL spectra of MoS_2_ without O_2_ and with *in situ* O_2_ processed samples respectively. (f) Schematic energy level diagram of conventional MoS_2_ (*i.e.* without O_2_) and oxygen-assisted MoS_2_ films.

The effect of adding oxygen was further investigated by photoelectron spectroscopy. X-ray photoelectron spectroscopy (XPS) demonstrate peaks corresponding to S 2s, Mo^4+^ 3d_5/2_ and Mo^4+^ 3d_3/2_ (indicating MoS_2_) at 226.7 eV, 229.6 eV, 232.8 eV, respectively (Fig. 4(a) and (b)). Along with the Mo^4+^ oxidation states, a small fraction of higher oxidation states was also observed, such as peaks at 233.6 eV and 235.9 eV corresponding to Mo^6+^ 3d_5/2_ and Mo^6+^ 3d_3/2_ respectively, for the *in situ* oxygen processed samples (Fig. 4(b)).^43,44^ These higher oxidation states could indicate molybdenum oxide formation, but their concentration is less than 7% (estimated from the fitted area of MoS_2_ and MoO_3_ oxidation), which could indicate their origin as surface contaminants.

The high crystallinity of oxygen-assisted MoS_2_ growth can be inferred from photoluminescence (PL) spectroscopy. Comparable to conventional MoS_2_, oxygen-assisted MoS_2_ shows a prominent peak near 1.8 eV which corresponds to the direct bandgap absorption of single-layer MoS_2_ (Fig. 4(c)).^45^ Surprisingly, an enhancement of PL peak intensity for oxygen-assisted CVD over conventional MoS_2_ by 15 times can be seen. This significant increase in quantum yield provides an exciting route towards improving MoS_2_ optoelectronic performance without the need for post-growth treatment.^46^ Detailed characterization of the PL spectra (Fig. 4(d) and (e)) demonstrates a decrease in the concentration of charged excitons (*i.e.* trions) and a subsequent increase of the neutral exciton emission for O_2_ processed samples. The schematic energy level diagram of conventional and oxygen-assisted MoS_2_ is shown in Fig. 4(f), where transitions of excitons and trions are shown.

To identify the reason for this difference, we conduct Kelvin probe measurements. The work function for individual triangle MoS_2_ samples (O_2_ free) was 4.34 eV, whereas the work-function of *in situ* oxygen processed samples was increased to 4.73 eV. The increase of work function in *in situ* O_2_ processed samples indicates that the usually n-type material is rendered more neutral.^47^ This effect could be due to the decrease in sulfur vacancies that cause n-type doping.^48^

These results indicate that the enhanced quantum yield's origin is a decrease in nonradiative recombination through trion states brought about by a lowered defect-induced doping.^49^

**Fig. 5:**
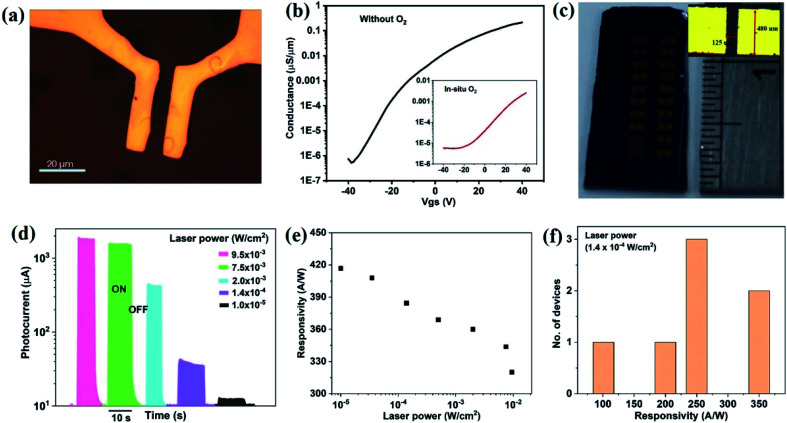
(a) Optical microscopy image of MoS_2_ back gate field effect transistor with channel length 6 μm (scale bar 20 μm). (b) Transfer characteristic of individual triangular MoS_2_ domains with fixed bias voltage (*V*_ds_) 10 V (for continuous MoS_2_ films, transfer characteristic shown in inset). Photosensor characteristics of *in situ* O_2_ processed continuous MoS_2_ films; (c) camera image of large area MoS_2_ photosensor array in centimetre dimension (channel length = 125 μm), (d) photocurrent variation with different laser power, (e) responsivity of the device with different laser power, (f) average responsivity of the MoS_2_ films shown at fixed power of 1.4 × 10^−4^ W cm^−2^.

We illustrate the potential of the increased scale and quality of MoS_2_ growth by producing electronic device arrays. As a first example, transistor devices were fabricated using photo-lithography (Fig. 5(a)) (more details on the device fabrication can be found in the ESI S2†). The transfer curves of oxygen-processed MoS_2_ show a higher threshold voltage than conventionally grown MoS_2_, which corroborates the observed decrease in n-type character (Fig. 5(b)). Notably, the transistor channel for O_2_ grown MoS_2_ includes many polycrystalline regions, whereas the conventional grown MoS_2_ transistor was designed around a single-crystalline flake (Fig. 5(a)), necessitating complex alignment schemes. Consequently, the observed transfer characteristics of the O_2_-grown MoS_2_ represent an averaging of the conductivity from the dozens of individual flakes and their boundaries. Despite this disadvantage, the transfer characteristics of large scale O_2_ grown MoS_2_ exhibits a similar behaviour as microscopic MoS_2_ crystals, indicating the good connectivity between polycrystalline regions. The measured field-effect mobility for both device types is within the range 0.1–10 cm^2^ V^−1^ s^−1^ of previous results in the literature for the similar structure of back gated MoS_2_ field-effect transistor devices.^50,51^

As a second application of MoS_2_ high optoelectronic performance, we produce centimeter-scale photosensor arrays (Fig. 5(c)). A variable photocurrent (*I*_ph_) was observed when toggling illumination with different laser power (Fig. 5(d)). The responsivity (*R*) of the device, defined as the ratio of photocurrent to the incident power (*P*_in_), increases with decreasing laser power (Fig. 5(e)) and reaches values in excess of ∼420 A W^−1^ in two-terminal co-planar geometry. This value represents the quite higher responsivity of MoS_2_ as compared to other reports^11,41,52,53^ (for a comparison to literature, see the ESI S3†) and indicates the benefits of producing MoS_2_ with low trap density and efficient carrier conduction. More importantly, uniformly high responsivity could be achieved for all devices throughout the centimetre-sized samples (Fig. 5(f)). The stability of photosensor also performed using *I*–*T* measurement for 10 cycles and details are given in the ESI S4.† In the support of large scale photodetector array, a uniform photosensitivity of MoS_2_ on Si/SiO_2_ is shown in ESI S5,† where, wafer scale photosensitivity is demonstrated (Table 1).

**Table 1:** Responsivity data comparison for MoS_2_-based photosensors

Responsivity (A W^−1^)	Wavelength and laser power	Area of devices (*L*/*W*) (in μm)	Materials	References
1 at *V*_ds_ = 1.5 V	532 nm, 200 μW	A few micron/tens to 100 micron	CVD MoS_2_	1
1 × 10^−3^ at *V*_ds_ = 1 V	405 nm, 100 μW	2/20	CVD MoS_2_	2
59 at *V*_ds_ = 1.2 V	532 nm, 1.69 × 10^−3^ W cm^−2^	5/∼30	Exfoliated MoS_2_	3
780 at *V*_ds_ = 1 V	532 nm, 1.3 × 10^−4^ W cm^−2^	—	CVD MoS_2_	4
1.1 × 10^6^ at *V*_ds_ = 0.15 V	460 nm, 0.33 pW	—	Exfoliated MoS_2_	5
1.1 × 10^−3^ at *V*_ds_ = 1.5 V	514.5 nm, 1 μW	0.8/5	CVD MoS_2_	6
0.42 × 10^−3^ at *V*_ds_ = 1 V	550 nm, 80 W cm^−2^	2.1/2.6	Exfoliated MoS_2_	7
7 at *V*_ds_ = 1 V	488 nm, 1 μW	—	CVD MoS_2_	8
420 at *V*_ds_ = 15 V	532 nm, 10^−5^ W cm^−2^	125/480	CVD MoS_2_	**This work**

## Conclusion

Continuous single-layer MoS_2_ films on Si/SiO_2_ substrates were successfully fabricated through an oxygen-assisted CVD growth process. Modification of the precursor transport resulted in highly crystalline material with well-interconnected domains. The absence of defects was shown to decrease the n-type doping of MoS_2_ and enhance its optoelectronic performance. Large array of highly sensitive photosensors were produced that exhibit uniform and 200-fold increased responsivities over the entire substrate as compared to other reports. The presented combination of increased MoS_2_ quality, scale, and commercial appeal open up new routes towards the future of 2D materials.

## Experimental section

### MoS_2_ synthesis

MoS_2_ thin films on Si/SiO_2_ were prepared by a CVD process, where 3 inch quartz tube furnace, having three different heating zone, was used. Si/SiO_2_ substrates were cleaned by sonication in acetone for 15 min, and then isopropyl alcohol followed by water wash and then dry with N_2_ gun. MoO_3_ powder (20 mg) was placed into two alumina boat (10 mg in each) at the lower heating zone furnace, which was upstream relative to center zone furnace. H_2_S gas (99% diluted with Ar) 400 sccm was used as a source of sulfur. It also acts as a carrier gas for MoO_3_ vapor to react with H_2_S and get deposited on the substrate. Separate Ar gas of 100 sccm was also used in the process. Substrate was placed at the center zone furnace facing polished surface upside and kept at a distance of 20 cm downstream relative to MoO_3_ powder. MoS_2_ films were deposited for 40 min at a low process pressure of 4 torr. Substrates were placed at 900 °C in the centre zone furnace, while MoO_3_ powder were placed at lower temperature of 750 °C. After purging the furnace with pure Ar for 1 h, furnace was heated to 600 °C at a heating rate of 30 °C min^−1^ and then maintained at the same temperature for 5 min. Further, temperature was increased to 900 °C at a rate of 30 °C min^−1^. A small amount of oxygen (0.5 sccm) was also added during the deposition. After completion, furnace was cooled down naturally. Here, we have only reported for the films deposited with O_2_ 0.5 sccm, because with higher oxygen rate the pressure inside the furnace was not stable and started to keep on increasing with increasing process time.

### MoS_2_ characterization

The surface morphology of the samples was characterized by AFM (model: Veeco, Dimension Icon) and TEM (model: JEOL, JEM-2100F) measurements. Raman spectra and PL measurements (model: HORIBA, Jobin Yvon Technology iHR 550) were recorded under ambient using a laser excitation wavelength of 532 nm. Laser spot size was ∼1 μm^2^ and laser power at the sample surface was 3 mW. Chemical configuration was characterized by XPS (model: ULVAC-PHI, PHI Quanterall), where XPS peak was calibrated with C 1s (284.6 eV). Work function of the sample surface was measured by Kelvin probe force microscopy (model: KP Technology Ltd., DCU series-10) using Pt coated tip in non-contact mode. The cantilever tip was calibrated using a standard Au plate electrode.

## Author contributions

M. S. synthesis of MoS_2_ films *via* CVD and also fabricated the devices and performed the electrical and optical measurements. M. S., Y. S.-C., and Z. L.-Y. analyzed Raman and AFM measurements. R. G. and M. S. performed the photosensor measurements and analyzed the responsivity data. Y. P.-H., Y. F.-C., and M. H. supervised the project. M. S., Y. P.-H., Y. F.-C., and M. H. have contributed to writing the paper through the input of all the authors.

## Conflicts of interest

There are no conflicts to declare.

## Supplementary Material

RA-012-D1RA06933K-s001
