# Supplementary material for: Chemical vapor deposition merges MoS2 grains into high-quality and centimeter-scale films on Si/SiO2
Source: RSC Adv. 2022 Feb 18;12(10):5990–6. doi: 10.1039/d1ra06933k (PMC8982092; doi:10.1039/d1ra06933k)
Supplement: RA-012-D1RA06933K-s001 [file RA-012-D1RA06933K-s001.pdf]

Electronic Supplementary Material (ESI) for RSC Advances.
This journal is © The Royal Society of Chemistry 2021

# Chemical vapor deposition merges $MoS_2$ grains into high-quality and centimeter-scale films on $Si/SiO_2$

Mukesh Singh[a], Rapti Ghosh[b,c,d], Yu-Siang Chen[c], Zhi-Long Yen[a], Mario Hofmann[a], Yang-Fang Chen[a] and Ya-Ping Hsieh[c,d†]

[a]Department of Physics, National Taiwan University, Taipei 106, Taiwan

[b]Department of Physics, National Central University, Chung Li 320, Taiwan

[c]Institute of Atomic and Molecular Sciences, Academia Sinica, Taipei 115, Taiwan

[d]Molecular Science and Technology, Taiwan International Graduate Program, Academia Sinica, Taipei 115, Taiwan

[†]Correspondence to: yphsieh@gate.sinica.edu.tw (Ya-Ping Hsieh)

**Supplementary data S1: Uniformity of the in-situ $O_2$-processed continuous $MoS_2$ films**

Uniformity of the $MoS_2$ films was measured using AFM and it showed ~1 – 2 nm thickness. Figure 1 represents the AFM images of different oxygen-assisted $MoS_2$ samples and its surface height profile, which confirms about the reproducibility as well as large area uniformity. To confirm the large area uniformity of the film thickness, we have measured the thickness at several locations (~ 10 places) using AFM and all measurements show the same result of ~ 1 – 2 nm. (Here, we have

shown the AFM image and corresponding surface height profile of a few places.) These results show that films are quite uniform in large area.

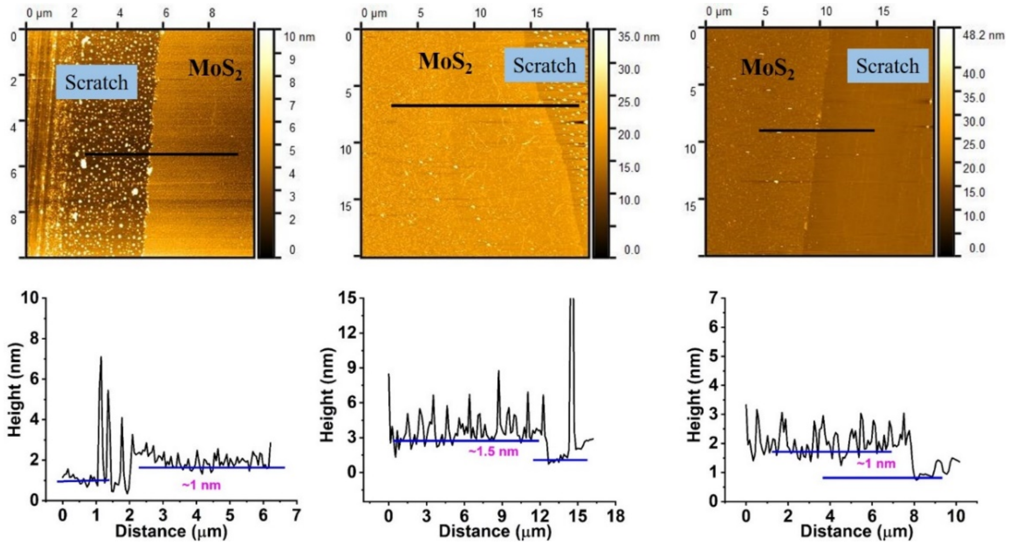


**Figure 1:** AFM images of different oxygen-assisted $MoS_2$ films and its surface height profile, which confirms about the reproducibility as well as large area uniformity.

**Supplementary data S2: Transistor device fabrication on Si/$SiO_2$**

Field effect transistor devices were fabricated on Si/$SiO_2$ (90 nm) using photolithography followed by thermal evaporation of Ti (3 nm) / Au (80 nm) for source and drain electrodes on top of $MoS_2$. Thermally grown 90 nm $SiO_2$ acts as gate dielectric and Si acts as gate electrode. The channel length of the device was 6 and 25 μm for the films processed without oxygen and in-situ oxygen samples respectively. After completion of lift-off process, devices were annealed in Ar environment at 200 $^{o}$C for 2h, for removing the organic residue. All the measurements were performed in ambient condition.

**Supplementary data S3: Responsivity data comparison for the two terminal co-planar geometry (in ambient condition)**

| Responsivity (A/W) | Wavelength and laser power | Area of devices (L/W)* (in µm) | Materials | References |
|---|---|---|---|---|
| 1 at $V_{ds}$ = 1.5 V | 532 nm, 200 µW | A few micron / tens to 100 micron | CVD $MoS_2$ | [1] |
| $1x10^{-3}$ at $V_{ds}$ = 1V | 405 nm, 100 µW | 2 / 20 | CVD $MoS_2$ | [2] |
| 59 at $V_{ds}$ = 1.2 V | 532 nm, $1.69x10^{-3}$ $W/cm^2$ | 5 / ~30 | Exfoliated $MoS_2$ | [3] |
| **780 at $V_{ds}$ = 1V | 532 nm, $1.3x10^{-4}$ $W/cm^2$ | --- | CVD $MoS_2$ | [4] |
| $1.1x10^6$ at $V_{ds}$ = 0.15V | 460 nm, 0.33 pW | --- | Exfoliated $MoS_2$ | [5] |
| $1.1x10^{-3}$ at $V_{ds}$=1.5V | 514.5 nm, 1 µW | 0.8 / 5 | CVD $MoS_2$ | [6] |
| $0.42x10^{-3}$ at $V_{ds}$=1V | 550 nm, 80 $W/cm^2$ | 2.1 / 2.6 | Exfoliated $MoS_2$ | [7] |
| 7 at $V_{ds}$=1V | 488 nm, 1 µW | --- | CVD $MoS_2$ | [8] |
| 420 at $V_{ds}$=15V | 532 nm, $10^{-5}$ $W/cm^2$ | 125 / 480 | CVD $MoS_2$ | **This work** |

* L is channel length; W is channel width. ** $V_g$-$V_{th}$ = 100V

CVD (Chemical Vapor Deposition)

**Supplementary data S4: I-T curve of oxygen assisted $MoS_2$ thin films**

For the stability of photosensor, I-T measurement were performed for 10 cycles with a laser power of $9.5x10^{-3}$ $W/cm^2$. A uniform sustainable of photocurrent for longer duration indicate that defect

density in oxygen-assisted $MoS_2$ is decreased, as shown in figure 2. Due to decrease in trap states, a fast recovery time of the order of millisecond is observed. The rise and fall time are 350 ms and 310 ms respectively.

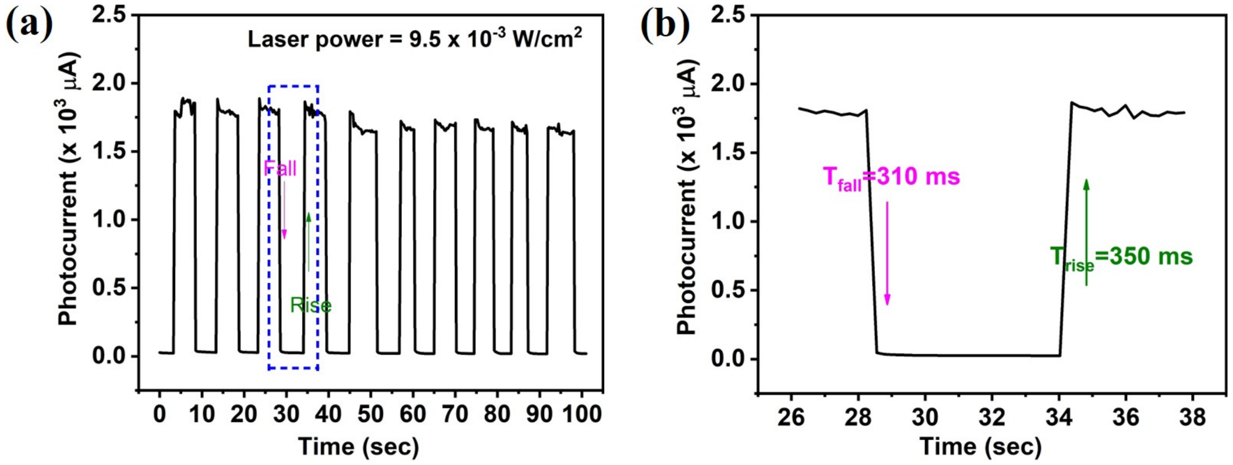


**Figure 2:** (a) I-T curve of oxygen assisted MoS2 thin films for 10 cycles at a fixed biasing of $V_{ds}$ 15V and (b) is corresponding fall and rise time 310 ms and 350 ms respectively.

**Supplementary data S5: Uniform wafer scale photosensitivity of $MoS_2$**

Wafer scale continuous $MoS_2$ films were grown on $Si/SiO_2$ using 3-inch CVD system. Figure 3 represents the large scale growth of $MoS_2$ in centimetre dimensions and its photoluminescence histogram around the whole wafer. Photoluminescence of the sample were measured randomly in one direction from one end to another end with a step of 2 mm. The histogram of photoluminescence intensity shows almost uniform single layer $MoS_2$ films over the entire substrate. However, a small decrease in photoluminescence intensity is observed at the other end of the samples. This may be due to the bilayer $MoS_2$, which could be possible due to the

concentration gradient of $MoO_3$ vapor in large area i.e. higher towards $MoO_3$ source and lower far away from the source.

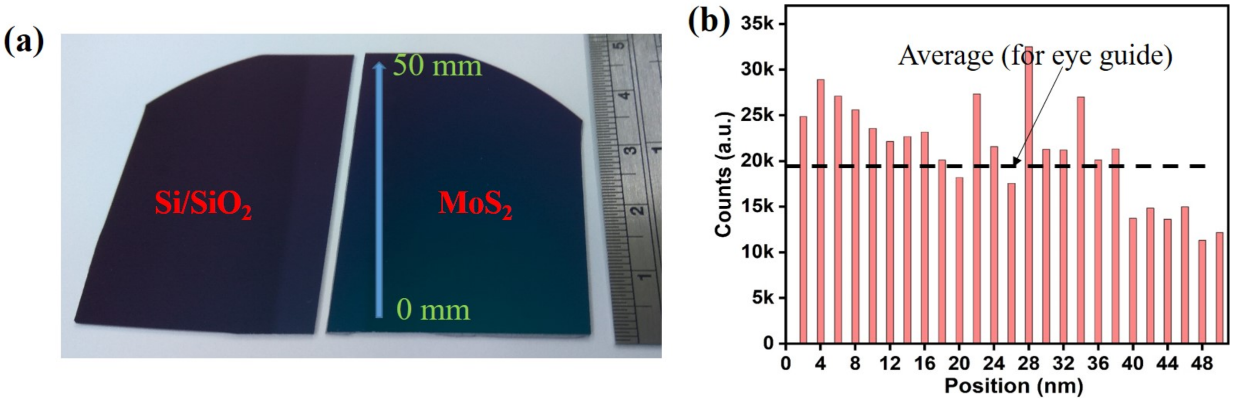


**Figure 3:** (a) Wafer scale continuous $MoS_2$ growth on Si/$SiO_2$ substrate (right side). A blank Si/$SiO_2$ substrate (left side) is shown here for the reference of contrast image. (b) Histogram of photoluminescence intensity measured on the wafer scale samples from one end to another end in one direction with 2 mm step. (Black dashed line is represented for eye guide only).

References

1 S. Khadka, T. E. Wickramasinghe, M. Lindquist, R. Thorat, S. H. Aleithan, M. E. Kordesch and E. Stinaff, *Applied Physics Letters*, 2017, **110**, 261109.

2 A. E. Yore, K. K. H. Smithe, S. Jha, K. Ray, E. Pop and A. K. M. Newaz, *Applied Physics Letters*, 2017, **111**, 043110.

3 W. Tang, C. Liu, L. Wang, X. Chen, M. Luo, W. Guo, S. W. Wang and W. Lu, *Applied Physics Letters*, 2017, **111**, 153502.

4 W. Zhang, J. K. Huang, C. H. Chen, Y. H. Chang, Y. J. Cheng and L. J. Li, *Advanced Materials*, 2013, **25**, 3456–3461.

5 R. Nur, T. Tsuchiya, K. Toprasertpong, K. Terabe, S. Takagi and M. Takenaka, *Communications Materials*, 2020, **1**, 103.

6 N. Perea-López, Z. Lin, N. R. Pradhan, A. Iñiguez-Rábago, A. L. Elías, A. McCreary, J. Lou, P. M. Ajayan, H. Terrones, L. Balicas and M. Terrones, *2D Materials*, 2014, **1**, 011004.

7 Z. Yin, H. Li, H. Li, L. Jiang, Y. Shi, Y. Sun, G. Lu, Q. Zhang, X. Chen and H. Zhang, *ACS Nano*, 2012, **6**, 74–80.

8 J. Jadwiszczak, G. Li, C. P. Cullen, J. J. Wang, P. Maguire, G. S. Duesberg, J. G. Lunney and H. Zhang, *Applied Physics Letters*, 2019, **114**, 091103.
